# AI-Based CCTV Monitoring of Road Drainage Inlet Blockage: Effects of Observation Height and Angle on Detection Performance

**DOI:** 10.3390/s26206552

**Published:** 2026-10-15

**Authors:** Hyunsuk Park, Aram Jung, Jinpyung Kim

**Affiliations:** 1 Department of Highway and Transportation Research, Korea Institute of Civil Engineering and Building Technology, Goyang-si 10223, Gyeonggi-do, Republic of Korea; hysupark@kict.re.kr; 2 School of Electronic Engineering, Kyonggi University, Suwon-si 16227, Gyeonggi-do, Republic of Korea; jinpkim@kyonggi.ac.kr

**Keywords:** drainage inlet blockage, camera installation geometry, occlusion robustness, condition assessment, urban flooding

## Abstract

Blockage of road drainage inlets by debris is a major cause of urban flooding. Manual inspection of urban drainage facilities is time-consuming and costly, highlighting the need for automated inspection technologies. In this study, a closed-circuit television (CCTV)- and AI-based technology was developed to automatically monitor the drainage inlet blockage status by varying camera height and observation angle to examine their influence on detection performance. The YOLOv11l and RT-DETR-L models classified the drainage inlet conditions in the testbed into three categories: fully open (clean), partially blocked (trash), and fully blocked (clogged). Both models achieved F1 scores of 0.994. Under clogged conditions, RT-DETR-L achieved a higher confidence score, whereas YOLOv11l achieved a slightly higher intersection over union (IoU). A three-way analysis of variance (ANOVA) revealed that the inlet condition was the most influential factor affecting both the confidence score and IoU, with a significant height–angle interaction under the clogged condition: performance improved with height at a front-facing angle but declined at oblique angles. These findings indicate that observation height and angle should be considered jointly when evaluating CCTV configurations for drainage inlet monitoring. Further validation using independent real-world CCTV data is required before extending these findings to broader field applications.

## 1. Introduction

In recent years, South Korea has experienced an increase in the frequency and intensity of localized heavy rainfall and linear rainbands owing to climate change, resulting in flooding in low-lying urban areas and alleyways. One cause of urban flooding is inadequate drainage. External debris, such as fallen leaves and litter, blocks drainage inlets and impedes rainwater drainage. Managing drainage facilities requires physical inspections to check their conditions and perform maintenance, such as debris removal. However, considering the vast number of drainage facilities in a city, it is time-consuming and cost-prohibitive for inspection personnel to check each facility. Therefore, there is a need to develop an automated inspection technology that can efficiently assess drainage facility conditions while minimizing the need for manual labor.

Accordingly, this study aims to develop a video-based automated inspection technology to replace manual inspection. Specifically, the objective is to develop an AI-based object detection technology that can classify drainage inlet conditions using existing security and multipurpose closed-circuit television (CCTV) systems installed by local governments. To this end, a road drainage facility testbed was constructed for data collection.

A literature review was conducted to select an object detection model and to identify the differentiating aspects of this study. Deep learning-based YOLO models have been studied for use in building object recognition models by pretraining on diverse images, with further training enabling the recognition of additional objects [1,2]. A previous study developed an illegally parked vehicle recognition model using YOLOv8 for object recognition and ResNet18 for object classification, achieving an F1 score above 0.8, thereby demonstrating that the proposed model had performance suitable for identifying illegally parked vehicles [3]. A study comparing the performance of five YOLO and RT-DETR models for detecting aerial objects in urban areas found that RT-DETR-r50 achieved the highest average precision, whereas YOLOv8n achieved the fastest inference speed [4]. Considering both speed and accuracy, YOLOv8s was found to be the most suitable model.

The review examines prior studies that detected and classified images of road facilities that were identical or similar to the drainage facilities targeted in this study. A study that detected curbside storm drains from Google Street View images using a Faster R-CNN model achieved an accuracy exceeding 80% [5]. A study developing a CNN-based model to detect small manhole-covered objects from high-resolution aerial imagery reported a maximum precision of 75% and a recall of 49% [6], indicating a false-positive rate of 25% for other objects misidentified as manholes and a false-negative rate of 51% for missed manholes. A study using stereo-depth camera imagery and a YOLO-based model to detect road manholes achieved a high detection accuracy of 96.6% [7]. Based on this literature review, image-based object classification models for this domain typically use CNN- or transformer-based architectures to achieve an accuracy of at least 75%. Accordingly, in this study, YOLOv11, a CNN-based model, and RT-DETR, a transformer-based model, were selected for drainage facility recognition and classification.

However, the image data used in previous studies to detect road drainage facilities were captured from fixed viewpoints, such as Google Street View or aerial imagery. In contrast, this study established scenarios for camera height and angle and further subdivided the degree of inlet blockage into three stages, thereby utilizing images of diverse blockage conditions captured from multiple viewpoints as the analysis dataset. This provides broader coverage of observation conditions than prior studies, as well as the added capability of classifying the blockage status. In addition, this study statistically examines the effects of external environmental factors, including camera height and angle, on the detection and classification performance of the models, thereby providing practical guidelines for the utilization of CCTV footage. A comparison of the dataset scale and environmental conditions with those of previous studies, along with the differentiating aspects of this study, is presented in Table 1.

This paper is organized into six sections. Section 1 (Introduction) presents the background and necessity of the research and reviews prior studies to select the AI models applied in this research. Section 2 (Literature Review) presents a review of existing literature that utilizes CCTV footage for drainage detection. Section 3 (Materials and Methods) presents the testbed construction, dataset preparation process based on preprocessing, model application, and performance evaluation metrics. Section 4 (Results) presents the results of applying the AI models, comparatively analyzes the model performance using the evaluation metrics, and statistically verifies the interaction effects between the observation height and angle through a three-way analysis of variance (ANOVA). Section 5 (Discussion) discusses the academic significance and implications of the derived results and Section 6 (Conclusions) presents conclusions and directions for future research based on the findings of this study.

## 2. Literature Review

Clogging of drainage structures has emerged as a critical issue in urban areas experiencing increased inflows of floating debris. If debris accumulates during heavy rainfall or flooding events, reducing drainage capacity, it can severely aggravate flood water levels [10]. Consequently, the need for external monitoring of drainage outlets has increased, leading to various studies utilizing equipment such as vision and radar sensors for drainage inlet detection.

Most existing research related to the development of drainage monitoring technologies and methodologies primarily focuses on internal drainage pipe monitoring to detect structural damage or defects [11,12]. This study reviews existing literature that utilizes CCTV footage for drainage debris detection, consistent with the approach proposed in this research. An existing study presented an automated drain inspection framework integrating a reconfigurable teleoperated robot with a CNN-based object detection model to address the hazards and inefficiency of manual inspection [13]. By leveraging robot-assisted navigation and transfer learning on custom datasets, this framework effectively detects and classifies drain blockages while providing reliable mapping and localization in complex environments. However, a limitation of this approach is its reduced applicability during real-time rainfall events. A comprehensive evaluation of defects—such as deposits, tree roots, ruptures, corrosion, and deformation—in wastewater and stormwater pipelines using CCTV inspection technology was conducted, demonstrating that applying targeted trenchless repair methods tailored to structural defect severity (RI levels) significantly enhances pipeline durability and extends service life by 20 years [14].

Existing studies that have developed robots for preventing drainage clogging focus on directly inspecting pipe conditions by inserting and navigating robots inside the drainage channels. In contrast, the objective of this study is to automatically detect clogging conditions on the exterior of drainage inlets during routine urban monitoring. The key distinction from prior research lies in eliminating the need for dedicated physical interventions—such as deploying radar, LiDAR, or robotics—thereby enhancing both time and cost efficiency.

## 3. Materials and Methods

This study consisted of two stages: (1) constructing the data collection environment and preprocessing the data and (2) applying the AI models and evaluating their performance. In the first stage, a testbed was constructed and experimental scenarios were designed to collect data by varying the observation height, observation angle, drainage cover type, and blockage conditions of the test drainage facility. Image data extracted from the CCTV footage were annotated with bounding boxes and class labels to construct a dataset for training the AI models. In the second stage, the dataset was applied to the AI models to infer the drainage inlet conditions from the images, and the inference performance of each model was evaluated. The model performance was assessed based on the confusion matrix, accuracy, confidence score, and intersection over union (IoU), and the feasibility of CCTV-based detection of drainage inlet conditions was examined. In addition, a three-way ANOVA was performed to statistically verify the interaction effects among the independent variables and identify the factors influencing model performance.

### 3.1. Testbed Construction and Experimental Scenarios

**Figure 1:**
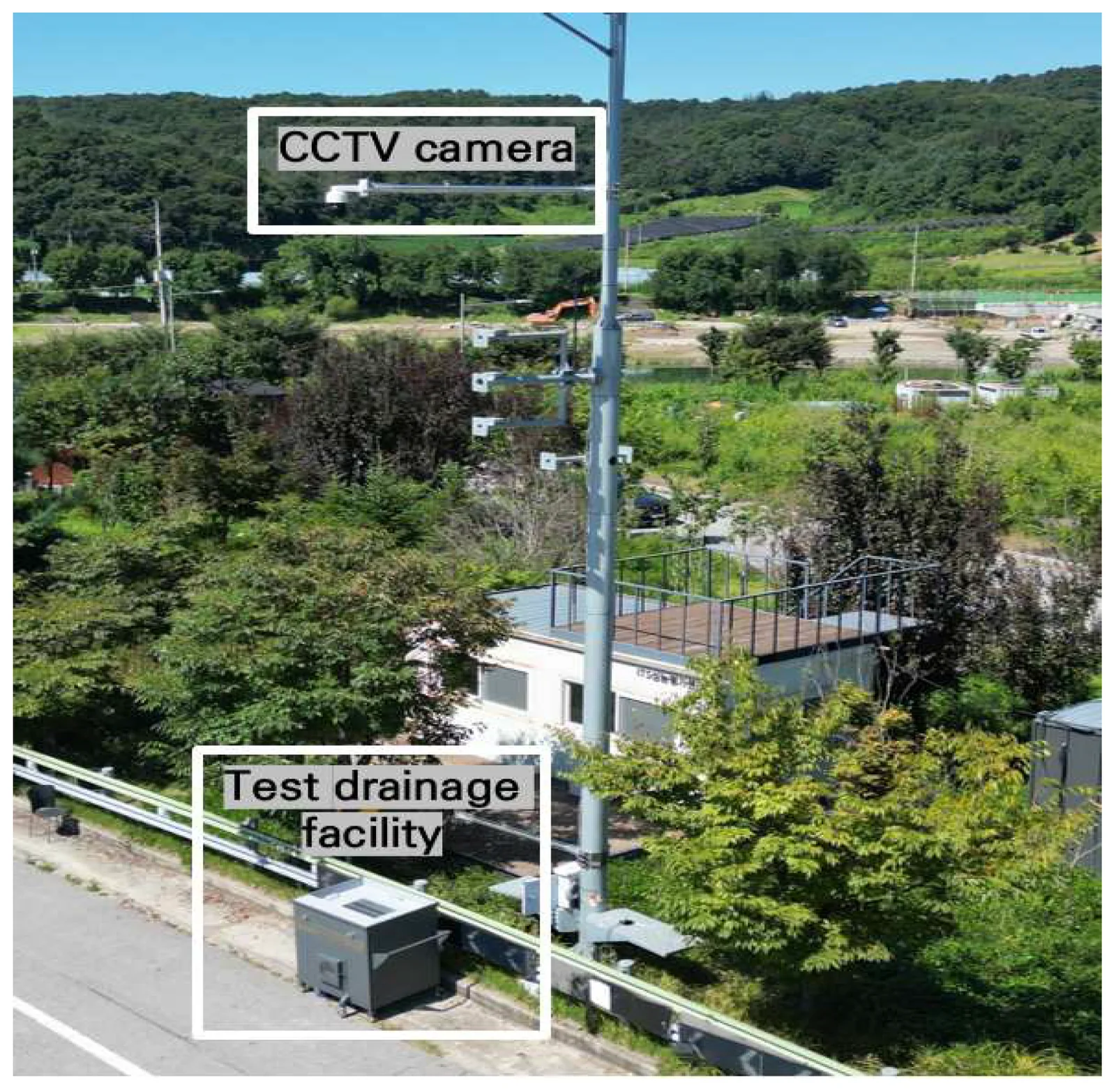
Testbed configuration.

To collect video and image data of road drainage facilities using a fixed CCTV camera, a test drainage facility and a CCTV camera (XNP-9300RWG, Hanwha Vision, Seongnam-si, Gyeonggi-do, Republic of Korea) were installed in a section of the Yeoncheon SOC Demonstration Center operated by the Korea Institute of Civil Engineering and Building Technology (KICT), and a testbed was constructed, as shown in Figure 1. Because the installation conditions of public CCTV systems, including the installation height and observation angle, vary depending on the surrounding environment, the CCTV mount was designed to allow height adjustment and pan/tilt control for implementing various fields of view. In addition, the test drainage facility was designed to allow cover replacement, relocation, and adjustment of debris height inside the sump. A transparent side panel was incorporated to enable visual monitoring of the internal conditions.

#### 3.1.1. Test Drainage Facility

**Figure 2:**
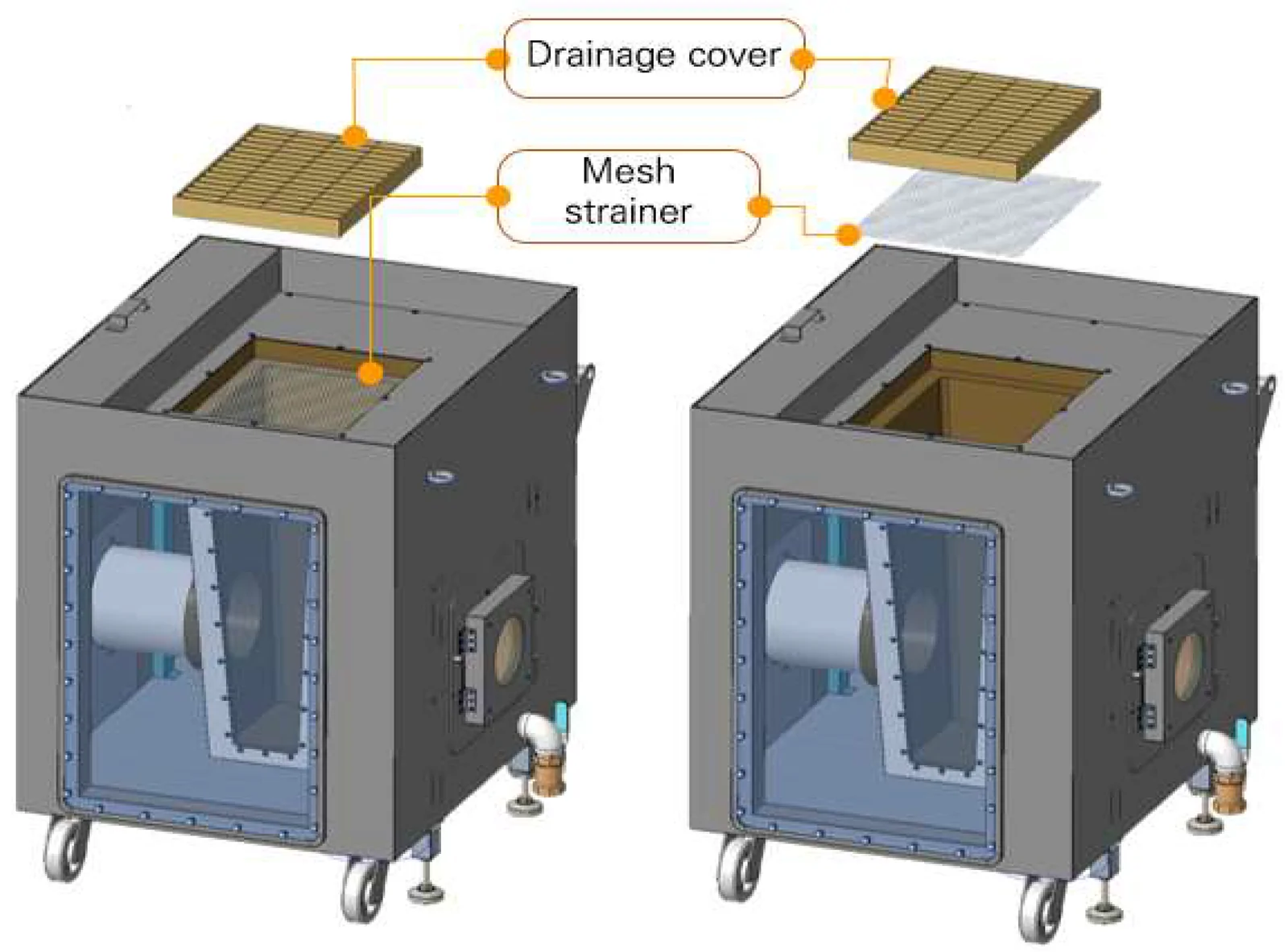
Design of the test drainage facility.

The test drainage facility used for the data collection was designed to replicate the specifications of an actual road drainage facility. It was fabricated as a movable enclosure equipped with a detachable cover. A transparent side panel was incorporated into the enclosure to monitor the internal conditions of the drainage facility. To reproduce realistic road conditions, the enclosure was manufactured in a dark gray color, similar to asphalt pavement, and a road curb was incorporated into the structure. The design of the test drainage facility is shown in Figure 2.

#### 3.1.2. Drainage Facility Covers

**Figure 3:**
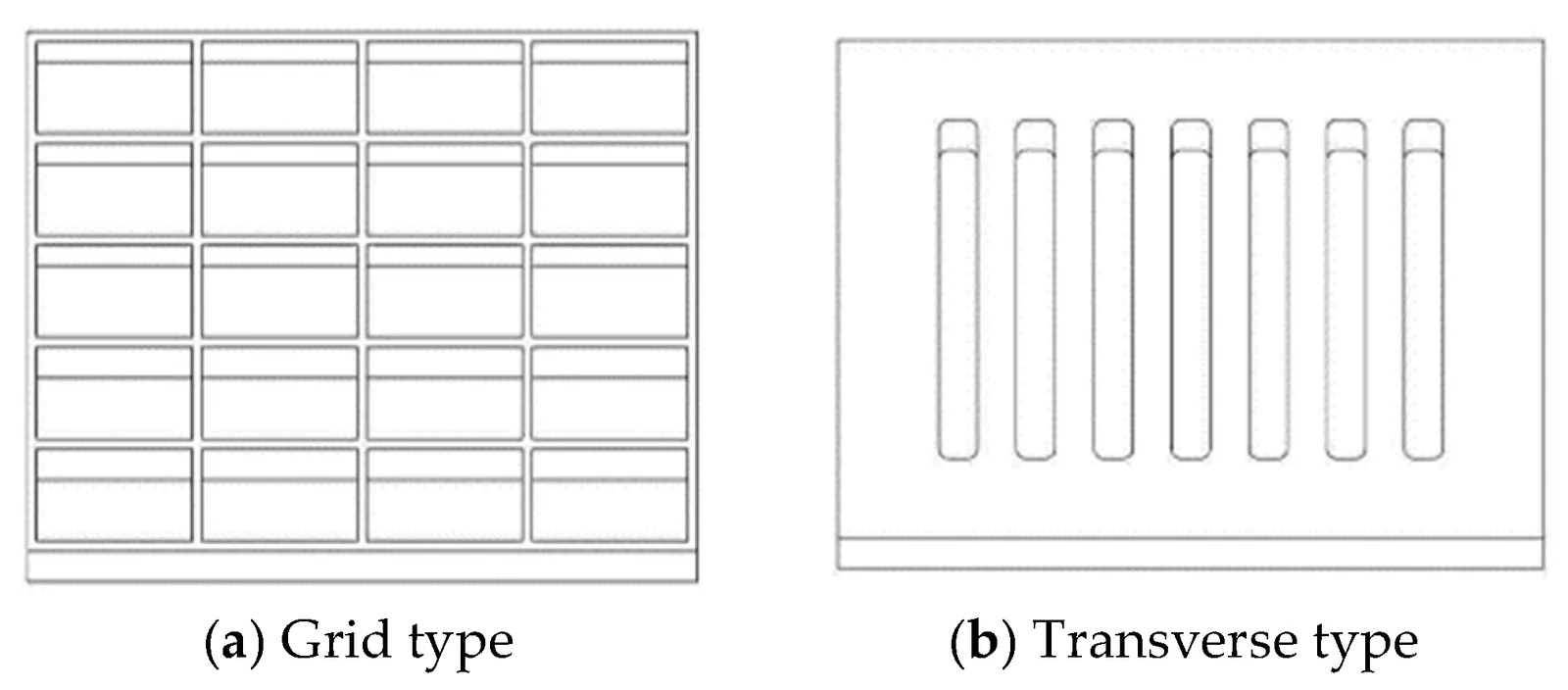
Drainage facility cover types specified in the management guidelines.

Road drainage facilities are equipped with various types of covers depending on the field conditions to ensure effective drainage. Therefore, different cover types may be installed, depending on the surrounding environment under actual road conditions. The grid- and transverse-type covers specified in the drainage facility management guidelines are shown in Figure 3. Suitable cover types for the experiment were selected based on these configurations.

**Figure 4:**
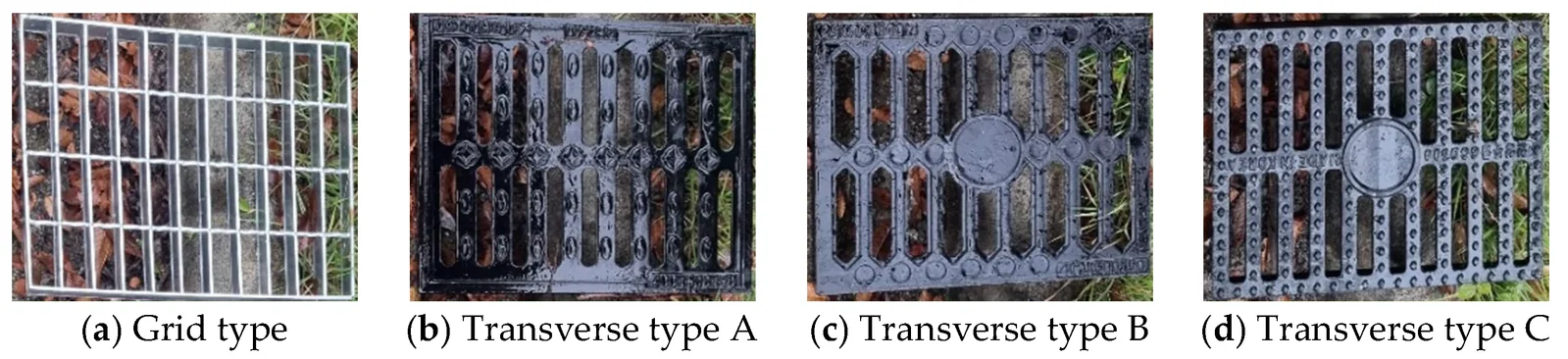
Types of drainage facility covers.

Commercially available drainage covers vary in shape and structure, and some differ from the types specified in the management guidelines. To ensure both experimental generality and field applicability of the developed technology, one grid-type cover and three transverse-type covers were selected. These covers followed the drainage hole configurations specified in the guidelines while considering various drainage opening sizes and cover structures with blocked central sections observed in actual field installations. The drainage covers used in this study are shown in Figure 4. The grid-type cover was made of steel and closely matched the shape specified in the management guidelines. The transverse-type covers A, B, and C, were made of ductile cast iron. Transverse Type A had no blocked sections in the middle and consisted of two rows of drainage openings, whereas transverse Types B and C had blocked central sections.

#### 3.1.3. Drainage Inlet Conditions

**Figure 5:**
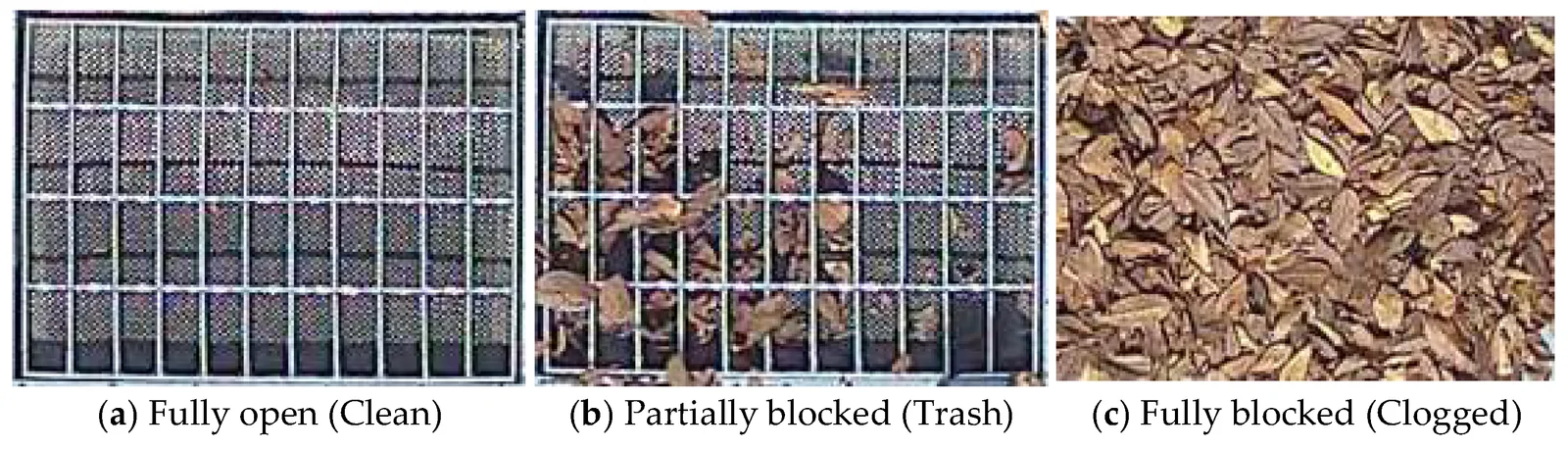
Drainage inlet condition scenarios.

In road environments, fallen leaves can accumulate in drainage facilities and obstruct drainage openings. To evaluate the AI-based detection performance when the drainage inlet was obscured, three conditions were defined, as shown in Figure 5. The first condition was a fully open condition (clean), in which the drainage cover was not blocked by leaves. The second was a partially blocked condition (trash), which was created by manually placing fallen leaves over the drainage inlet so that some of the drainage openings were obstructed while the inlet remained neither fully open nor fully covered. A predefined blockage percentage was not applied to this condition. The third condition was a fully blocked condition (clogged), in which the entire cover was covered by leaves and the drainage openings could not be visually identified.

#### 3.1.4. Observation Angle and Height of the Fixed CCTV Camera

**Figure 6:**
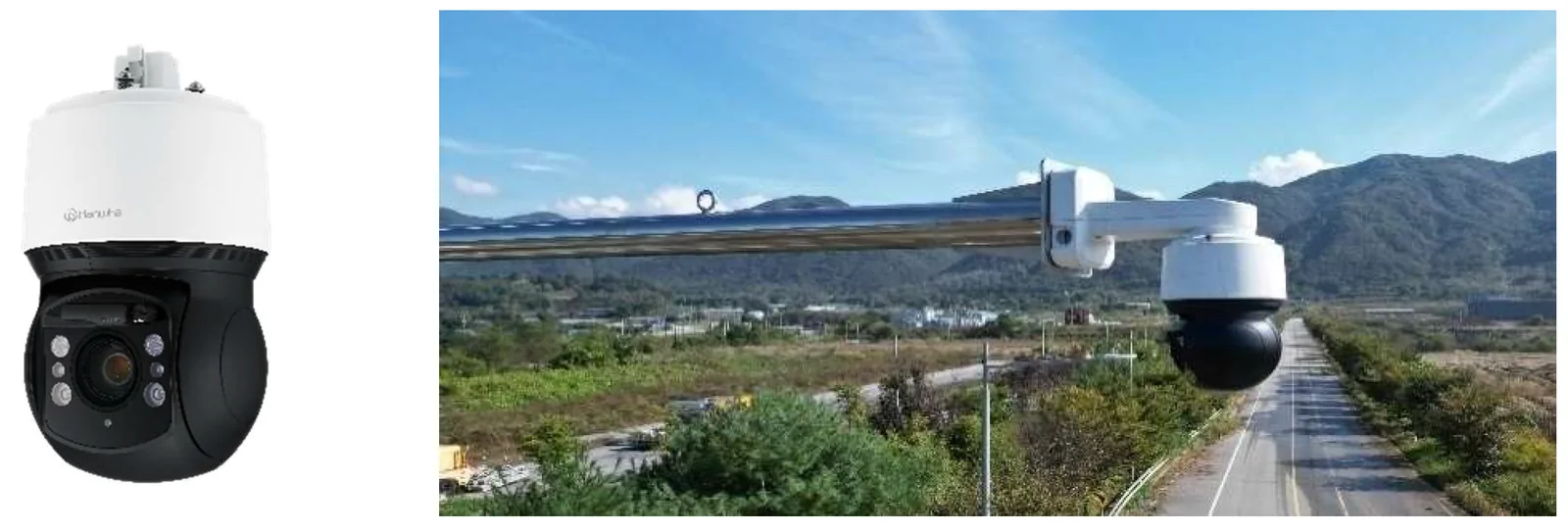
CCTV camera and installation configuration.

In South Korea, there are no explicit standards governing the installation height of fixed CCTV cameras. Instead, the installation height is determined comprehensively based on the purpose of installation (e.g., traffic monitoring, security, or multipurpose use), surrounding structures or obstacles at the installation site, and ease of maintenance. For traffic information collection under the ITS program, an installation height of 15 m is preferred to avoid obstruction by surrounding obstacles, whereas heights of 20–30 m are considered when an unobstructed view cannot be secured [15]. For cameras installed at the center of a road for traffic condition detection, a minimum height of 12 m is required [16]. CCTV cameras installed inside tunnels are mounted at heights of 3.5 m or higher to minimize the effects of lighting conditions and obstruction of the field of view by large vehicles [17]. For security and multipurpose CCTV systems, installation height is not fixed according to a single nationwide standard. Rather, it is determined based on local government specifications and site conditions, while complying with relevant personal information protection and installation/operation standards. Practically, installation heights of 4.5 m or higher above ground level are commonly applied. In this study, it was assumed that the CCTV camera was installed at a location where the field of view was not obstructed by surrounding obstacles (e.g., street trees) and did not interfere with nearby facilities (e.g., traffic signals). Accordingly, only the installation height and observation angle scenarios were established. Because road drainage inlets are relatively small objects on road surfaces, the object detection performance may be limited. Therefore, monitoring drainage conditions using footage from security and multipurpose CCTV cameras, which are generally installed at lower heights, is expected to provide more reliable detection results than using footage from traffic information CCTV cameras installed at greater heights. Accordingly, considering the practical installation height of security and multipurpose CCTV systems (4.5 m or higher) and the 1.3 m height of the test drainage facility, two CCTV installation height scenarios were established: 6 m and 10 m. The CCTV camera and its installation on the testbed are shown in Figure 6.

**Figure 7:**
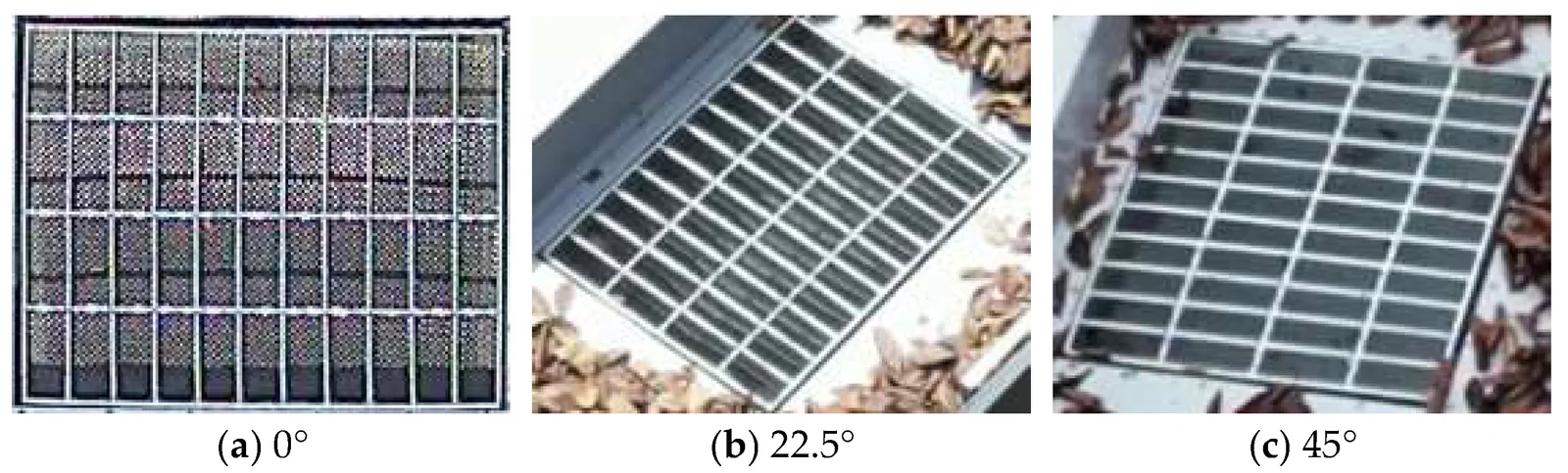
Detailed images of the drainage facility cover by observation angle.

For the observation angle of the drainage facility, three scenarios—0°, 22.5°, and 45° from the vertical axis—were established to analyze the changes in detection performance based on the observation angle. An observation angle of 0° represents the ideal condition in which the drainage facility is observed directly from above; however, this configuration makes it difficult to monitor the surrounding conditions. The 45° condition was selected as a representative oblique-view condition that allows the surrounding area to be observed together with the drainage inlet. The 22.5° condition was selected as the midpoint between 0° and 45° to examine changes in detection performance with increasing observation angle. These three angles were selected as controlled experimental conditions and were not intended to represent the full range of observation angles used in actual CCTV installations. Example images of the drainage facility at each observation angle are shown in Figure 7.

### 3.2. Collected Data

Video footage of the road drainage facility was collected across 72 experimental scenarios corresponding to combinations of two observation heights, three observation angles, three drainage inlet conditions, and four drainage cover types. A total of 38,239 frame-level images were extracted from the collected video footage. Because consecutive video frames contained a large number of visually similar or redundant images, 20 representative images were selected from each of the 72 experimental scenarios while minimizing visual redundancy. This resulted in a balanced dataset of 1440 images, with equal representation of the experimental conditions. Example images based on the observation heights and angles are presented in Table 2. The selected images were annotated with bounding boxes and class labels to train and evaluate the object detection models. Because the annotations were not independently duplicated and inter-annotator agreement was not quantified, some annotation bias, particularly for the partially blocked (trash) condition, cannot be ruled out. The dataset was then randomly divided at an 8:2 ratio into 1152 training images and 288 evaluation images. Based on the 72 experimental scenarios, the evaluation dataset consisted of an average of four images per scenario. Although the number of evaluation images was relatively limited, the factorial combination of observation height, observation angle, drainage inlet condition, and cover type enabled systematic evaluation across representative installation and blockage scenarios. Because the training and evaluation images were derived from the same controlled testbed and video acquisition environment, the reported performance should be interpreted as same-scene, in-domain performance rather than as independent real-world generalization.

### 3.3. AI Model Implementation

YOLOv11 and RT-DETR were selected as the state-of-the-art object detection models for detecting drainage inlets and classifying their blockage conditions using the collected image data. The YOLO model predicts object locations and classes simultaneously within a single neural network, providing advantages such as fast processing speed and high detection accuracy for object detection and classification tasks [18]. The YOLOv11l model incorporates architectural improvements and an optimized training strategy, achieving a balance between detection accuracy and computational efficiency. In addition, it can be effectively applied to various environments, including edge devices and cloud-based platforms [19]. RT-DETR was proposed to overcome the limitations of conventional DETR models. It reduces the computational complexity while maintaining high accuracy and the advantages of a transformer-based architecture, enabling real-time object detection [20]. In this study, YOLOv11l and RT-DETR-R50(L) (hereafter referred to as RT-DETR-L) were selected because the primary objective was to evaluate higher object detection performance under different CCTV observation conditions rather than to minimize model size. Accordingly, the large variants were used for the experiments, while smaller variants were not evaluated. The same training settings were applied to YOLOv11l and RT-DETR-L, except for the model-specific pretrained weights. In this study, YOLOv11l and RT-DETR-L were trained under the same experimental training configuration using the Ultralytics framework, with their respective pretrained weights (yolo11l.pt and rtdetr-l.pt). Both models were trained with an input image size of 640 × 640 pixels, a batch size of 4, and a maximum of 100 epochs. The initial learning rate (lr0) was set to 0.01, the final learning rate factor (lrf) to 0.01, and the optimizer was automatically selected. A weight decay of 0.0005 and a warm-up period of 3 epochs were applied. Early stopping was used with a patience of 50 epochs, such that training was terminated if the validation performance did not improve for 50 consecutive epochs.

Training-time data augmentation included color variation, translation, scaling, horizontal flipping, and mosaic augmentation. No additional offline augmentation was applied beyond the augmentation procedures configured within the training framework.

### 3.4. Accuracy Evaluation of AI-Based Road Drainage Facility Condition Assessment

#### 3.4.1. F1 Score

The F1 score is a metric used to evaluate the classification performance of the AI models and represents the harmonic mean of precision and recall. Precision indicates the proportion of instances predicted as positive by the model that are actually positive, thereby evaluating the correctness of the positive predictions. Recall indicates the proportion of actual positive instances correctly identified as positive by the model, thereby evaluating the completeness of the positive predictions. The F1 score uses the harmonic mean of precision and recall such that the overall score decreases when either precision or recall is low. A high F1 score indicates that the model achieves a balanced performance between precision and recall without being biased toward either metric. The equation for the F1 score is shown in Equation (1).

$$Precision=\frac{TP}{TP+FP}$$

$$Recall=\frac{TP}{TP+FN}$$

(1) $$F1=2\times \frac{Precision\times Recall}{Precision+Recall}$$

TP: Predicted True when actually True.

FP: Predicted True when actually False.

FN: Predicted False when actually True.

The F1 score reported in this study is the macro F1 score, which is calculated by averaging the F1 scores obtained independently for each of the three classes: fully open (clean), partially blocked (trash), and fully blocked (clogged).

#### 3.4.2. Confidence Score

The confidence score represents the reliability of the AI model predictions and indicates the probability that a particular prediction for an individual input image is correct, expressed as a value between zero and one. A higher confidence score indicates a higher probability that the AI model correctly predicts the drainage inlet conditions for the corresponding image. A confidence score was obtained from the predicted output generated by the AI model. The equation for the confidence score is shown in Equation (2). Because YOLOv11l and RT-DETR-L have different architectures and output mechanisms, their absolute confidence scores are not necessarily calibrated on the same probabilistic scale and therefore are not directly comparable. Accordingly, the confidence scores were interpreted primarily in terms of relative changes within each model across observation conditions.

(2) $$P_{i}=\frac{e^{z_{i}}}{\sum_{j=1}^{K}e^{z_{i}}}$$

$P_{i}$: Probability of class i.

$z_{i}$: Logit (linear output) of class i.

K: Total number of classes.

#### 3.4.3. Intersection over Union (IoU)

The IoU represents the degree of overlap between the bounding box predicted by the AI model and the ground-truth bounding box and is a metric used to evaluate object detection performance. The IoU was calculated as the ratio of the overlapping area between the predicted bounding box and the ground-truth bounding box to the union area of the two regions, resulting in a value between zero and one. An IoU value of zero indicates that there is no overlap between the predicted and actual regions, whereas a value approaching 1 indicates a greater agreement between the predicted and ground-truth bounding boxes. The equation for IoU is presented in Equation (3).

(3) $$IoU=\frac{Area\;of\;overlap}{Area\;of\;Union}$$

In this study, a detection result was considered a true positive when the IoU between the predicted bounding box and the ground-truth bounding box of the same class was 0.5 or higher. This threshold was used as the criterion for successful object detection.

### 3.5. Three-Way Analysis of Variance (ANOVA)

This study aimed to determine whether environmental factors, including observation height, observation angle, and drainage inlet conditions, individually affect the detection performance of AI models, and whether specific combinations of these factors cause additional changes in model performance. To achieve this objective, a three-way ANOVA was conducted to simultaneously evaluate the main effects and interaction effects among the three independent variables.

One-way ANOVA is a statistical method used to determine whether the differences in mean values between two or more groups are statistically significant. As this study considered three independent variables (observation height, observation angle, and drainage inlet conditions), a three-way ANOVA was applied. This method evaluates both the main effect of each independent variable on the dependent variable and the interaction effects caused by combinations of independent variables. The main effect represents the influence of a single independent variable on the dependent variable, regardless of changes in other independent variables. The interaction effect represents the extent to which the influence of one independent variable on the dependent variable changes, depending on the level of another independent variable.

If the *p*-value obtained from the statistical analysis was greater than or equal to the significance level, the difference in the dependent variable caused by the corresponding independent variable was considered statistically insignificant, indicating that the variable did not have a statistically significant effect on the dependent variable. Conversely, if the *p*-value was lower than the significance level, the difference was considered statistically significant, indicating that the corresponding variable affected the dependent variable. In this study, a significance level of 0.05, corresponding to a 95% confidence level, was adopted to determine whether the independent variables had statistically significant effects on the model performance.

## 4. Results

The location of the drainage facility was determined by applying the analysis dataset to the AI models, and the blockage status of the drainage inlet was inferred. Example results obtained by applying images captured at a height of 6 m to the YOLOv11l and RT-DETR-L models are presented in Table 3. For each image, the model detected the drainage inlet, determined its blockage condition, and output the F1 score, confidence score, and IoU. In Table 3, CA (Classification Accuracy) is used as an image-level indicator to determine whether the predicted class of the drainage inlet condition matches the ground-truth class. It assumes a value of 1.00 when the predicted and ground-truth classes are identical and 0 when they are different. Individual detection was classified as a true positive when CA was 1.00 and IoU was 0.5 or greater, and as a false positive otherwise. These classifications were aggregated across the entire evaluation dataset to compute the precision, recall, and F1 score reported in Table 4.

### 4.1. Object Detection and Classification Results

Table 4 presents the class-wise precision, recall, and F1 scores for the clean, trash, and clogged conditions, together with the macro-averaged results. Both models achieved consistently high performance across all three classes. YOLOv11l achieved a macro precision of 0.992, recall of 0.997, and F1 score of 0.994, whereas RT-DETR-L achieved corresponding values of 0.993, 0.996, and 0.994. The class-wise F1 scores ranged from 0.993 to 0.996 for both models, indicating that the high overall F1 score was not dominated by a particular drainage inlet condition.

Both YOLOv11l and RT-DETR-L showed consistently high performance across the clean, trash, and clogged classes, with no pronounced class-specific performance degradation. The macro F1 score was 0.994 for both models. In addition, both models achieved an mean average precision at 0.5 of 0.995, while the mean average precision at 0.5:0.95 was 0.966 for YOLOv11l and 0.952 for RT-DETR-L. These results indicate comparable overall detection performance, with YOLOv11l showing slightly better localization performance under stricter IoU thresholds. However, these results should be interpreted as same-scene, in-domain performance rather than evidence of independent field generalization.

### 4.2. Results of Confidence Score

#### 4.2.1. Characteristics of Confidence Score with Respect to Factor Variation

**Figure 8:**
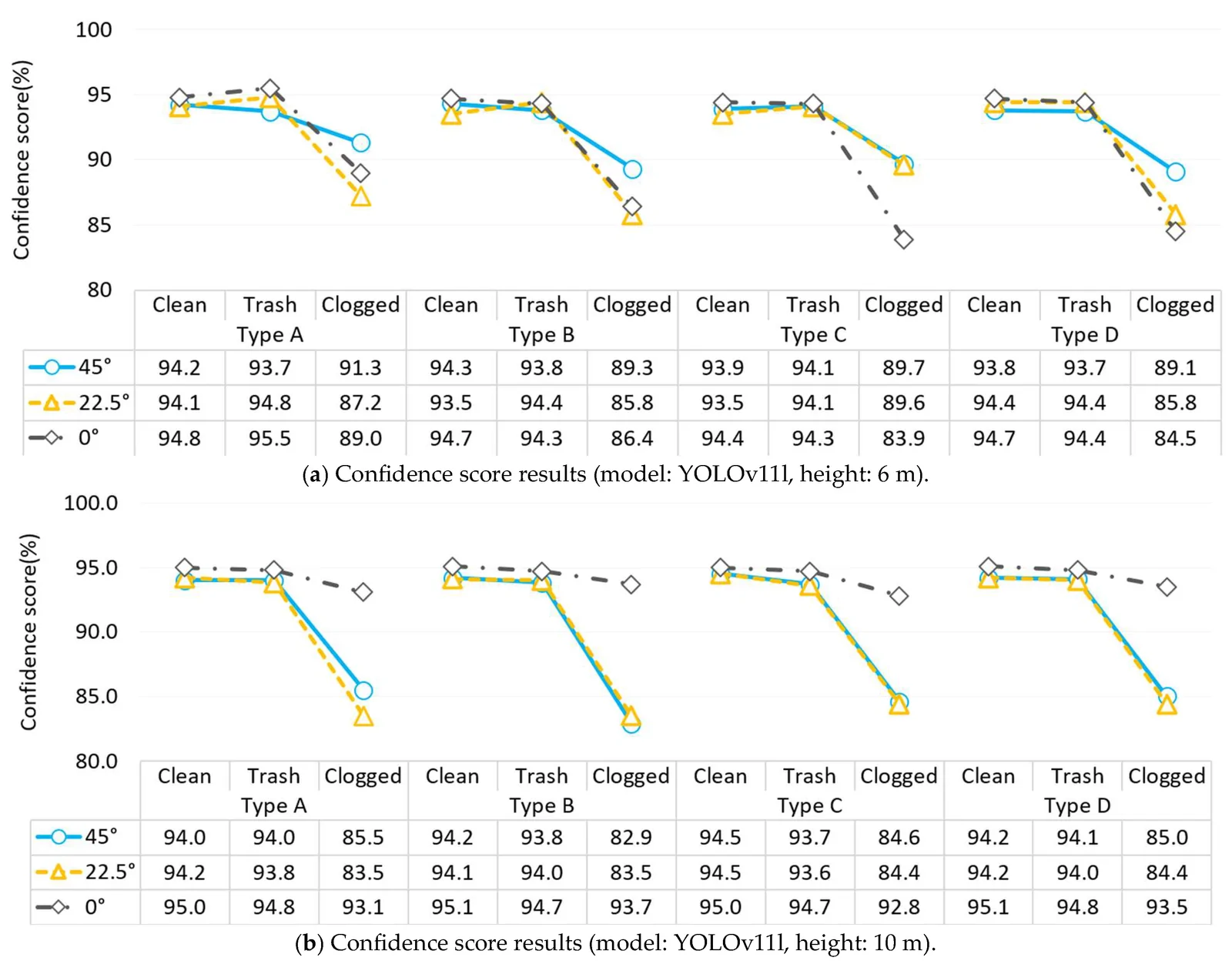

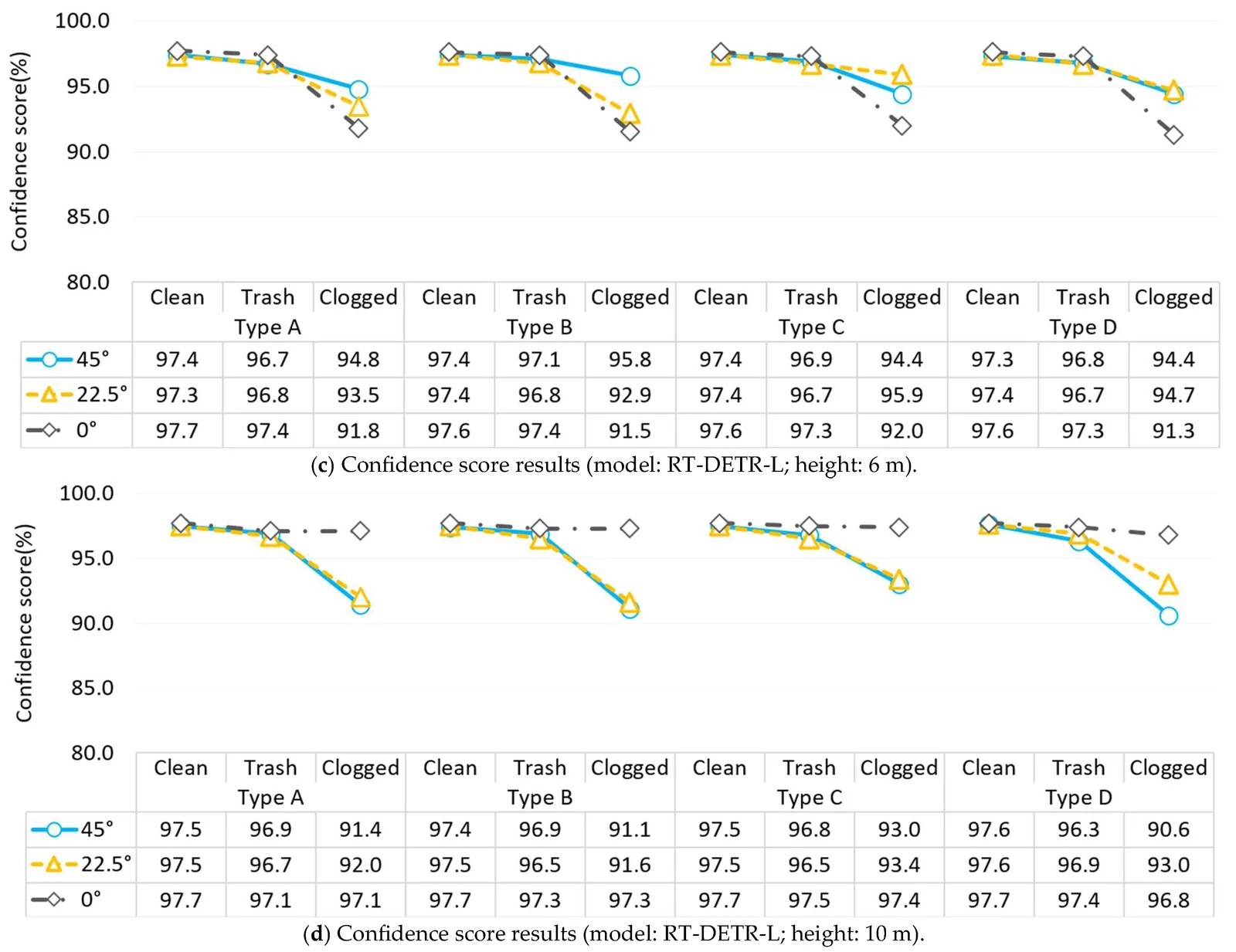
Confidence score results.

To investigate how the prediction confidence of the AI models varies with environmental factors (observation height, observation angle, and inlet condition), the confidence scores of the YOLOv11l model are presented as percentages in Figure 8a,b. Across all drainage cover types, the confidence scores exhibited different trends depending on the drainage inlet condition and the CCTV observation height and angle. At both observation heights (6 and 10 m), the confidence score was lower under the clogged condition than under the clean or trash conditions. For the clean and trash conditions, the confidence score showed a slight increase when the inlet was observed directly above (0°) compared with the side-view observations (22.5° and 45°). By contrast, under the clogged condition, the confidence score decreased regardless of the observation height or angle. At an observation height of 6 m, the confidence score obtained from the frontal view (0°) was lower than or similar to that obtained from the side views, whereas at 10 m it became higher than that obtained from the side views. Thus, under clogged conditions, the effect of the observation height differed depending on the observation angle. In particular, the confidence score increased markedly with increasing observation height under the frontal-view condition (0°), whereas it decreased slightly under the side-view conditions (22.5° and 45°).

The confidence scores of the RT-DETR-L model are presented as percentages in Figure 8c,d. At both observation heights, the confidence scores decreased in the order clean > trash > clogged. Under the clogged condition and frontal-view observation (0°), the effect of the observation height showed the opposite tendency, depending on the viewing angle. At the observation height of 6 m, the confidence score obtained from the frontal view was lower than that obtained from the side views and remained below 95%, whereas at 10 m, it exceeded 95% and was higher than that obtained from the side views. Thus, similar to YOLOv11l, the influence of the observation height under clogged conditions varied based on the observation angle. The confidence score increased markedly with increasing observation height under frontal-view observation but decreased slightly under side-view observation.

Under the clean and trash conditions, the characteristic features of the drainage cover remained visible in the images. In contrast, under clogged conditions, these features were largely obscured by fallen leaves, making it more difficult for AI models to recognize the drainage cover characteristics. The degree of difficulty varied depending on the observation conditions, and both models exhibited opposite trends for frontal- and side-view observations (the underlying reason is discussed in Section 4.2.5). Despite the reduction in confidence score under the fully blocked (clogged) condition, the minimum confidence score observed across both models was 82.9% (YOLOv11l at an observation height of 10 m), indicating that the confidence of the predicted drainage inlet conditions remained sufficiently high for practical applications.

#### 4.2.2. ANOVA Results for Drainage Facility Cover Type by Confidence Score

For the RT-DETR-L model dataset, although the Shapiro–Wilk test yielded p<0.001, the normality assumption was satisfied based on the skewness (−1.393) and kurtosis (0.634) criteria for large samples (N=1440). Homoscedasticity was also confirmed (Levene’s F=0.785,p=0.503). A one-way ANOVA revealed no statistically significant differences in CS across the Grid groups ($F\left(3,1436\right)=0.663,p=0.575,\eta^{2}=0.001$). These results indicate that the Grid factor had no significant impact on the performance of the RT-DETR-L model.

For the YOLOv11l model, the normality assumption was violated due to skewness (−3.142) and kurtosis (27.798), although homoscedasticity was satisfied (Levene’s F=0.603,p=0.613). Consequently, a nonparametric Kruskal–Wallis test was conducted. The results revealed no statistically significant differences in CS across cover types ($F=2.378,p=0.498,\eta_{H}^{2}=0.000$), indicating that the cover type had a negligible effect on performance in the YOLOv11l model.

Table 5 summarizes these results for both models. Neither model exhibited a statistically significant Grid effect on CS, and the corresponding effect sizes were negligible in both cases (η^2^ ≤ 0.001), indicating that cover type accounted for essentially none of the variance in CS. Because neither main effect reached statistical significance, post hoc multiple comparison tests were not conducted for either model.

#### 4.2.3. Statistics by Confidence Score

To quantitatively compare the performances of the two AI models, the descriptive statistics of the confidence score according to the observation height and inlet conditions are presented in Table 6. Under clean and trash conditions, both YOLOv11l and RT-DETR-L achieved mean confidence scores (>94%) with minimal variability across heights. By contrast, in the clogged condition, the mean confidence score decreased, whereas the standard deviation increased. In YOLOv11l model, the standard deviation doubled between 6 m and 10 m (2.2 to 4.3). Overall, the mean confidence score of RT-DETR-L was approximately 3–6 percentage points higher than that of YOLOv11l. This difference was most pronounced under clogged conditions, where RT-DETR-L showed a confidence score approximately six percentage points higher than YOLOv11l. These findings indicated that RT-DETR-L showed higher prediction confidence and greater stability in confidence scores with respect to changes in observation height.

Descriptive statistics according to the observation angle are presented in Table 7. Under clean and trash conditions, both models achieved high mean confidence scores (>93%) and small standard deviations (<0.5), indicating stable performance across the observation angles. However, under the clogged condition, the mean confidence score decreased and the standard deviation increased. In particular, under frontal-view observation (0°), the standard deviation of the YOLOv11l model reached 3.9, compared to 2.8 for RT-DETR-L, indicating a greater sensitivity to the observation angle. Overall, RT-DETR-L achieved mean confidence scores approximately 2.6–7.9 percentage points higher than those of YOLOv11l, with the largest differences observed under clogged conditions. These findings indicate that RT-DETR-L showed higher prediction confidence and greater stability in confidence scores with respect to changes in the observation angle.

#### 4.2.4. Three-Way ANOVA Results by Confidence Score

To evaluate the confidence score (CS) outputs of YOLOv11l, assumption tests for normality and homogeneity of variance were conducted across four candidate transformations. The result are presented in Table 8. Across all transformations, the normality assumption was severely violated, with Shapiro–Wilk statistics ranging from W = 0.128 to W = 0.795 (all *p* < 0.001), indicating a highly non-normal distribution likely attributable to a ceiling/floor effect inherent to bounded confidence scores. Homoscedasticity was likewise violated under every transformation. Among the candidates, the logarithmic transformation yielded the lowest Levene’s F-statistic (F = 2.890, *p* < 0.001), representing the smallest degree of variance heterogeneity relative to the raw data (F = 3.661) and the other transformations (Sqrt: F = 3.243; Box–Cox: F = 14.596, which notably worsened homoscedasticity despite improving the Shapiro–Wilk statistic). Accordingly, the log-transformed CS values were adopted for the subsequent three-way ANOVA, on the basis of minimizing variance inequality while acknowledging that neither assumption was fully satisfied [21].

For RT-DETR-L, the normality assumption was satisfied across all four transformations (Shapiro–Wilk W ranging from 0.773 to 0.942, all *p* < 0.001 due to large sample size sensitivity, but formally classified as “Satisfied” per the testing criteria), in contrast to YOLOv11l. However, homoscedasticity was violated under every transformation, with substantially larger Levene F-statistics than those observed for YOLOv11l (raw: F = 124.912; log: F = 125.116; sqrt: F = 125.071; Box–Cox: F = 44.662). The Box–Cox transformation yielded the lowest Levene’s F-statistic among the candidates, representing the greatest reduction in variance heterogeneity (approximately a 64% reduction relative to the raw data), and was therefore adopted (CS_BoxCox) for the subsequent three-way ANOVA despite the persistent, though comparatively mitigated, violation of homoscedasticity.

The three-way ANOVA results indicated that for both models, the main effect of facility status (Status) exerted the primary influence on detection performance, outweighing physical camera setup parameters (Height and Angle). Notably, both models exhibited a prominent three-way interaction (Status×Height×Angle), implying that optimal camera installation height and angle are highly dynamic and contingent upon the specific condition of the drainage facility.

The results demonstrated that all main factors including Status, Height, and Angle along with their respective interaction terms, achieved statistical significance at the level of p<0.001 for both YOLOv11l and RT-DETR-L.

Across both object detection models, contamination status (Status) emerged as the most influential factor on the outcome metric, with the clogged condition showing the most pronounced deviation. However, YOLOv11l demonstrated relative robustness to variations in camera angle and installation height, whereas RT-DETR-L exhibited markedly greater sensitivity to these geometric imaging conditions (Angle η^2^p: 0.087 vs. 0.567; three-way interaction η^2^p: 0.198 vs. 0.723). These findings suggest that, in practical deployment scenarios, sensitivity to camera angle and mounting height requires more rigorous validation for RT-DETR-L than for YOLOv11l. Post hoc tests were not conducted for the three-way ANOVA because the study focused on the magnitude of main and interaction effects rather than subgroup-level ranking; simple-effect analyses of the significant interactions remain a limitation.

#### 4.2.5. Interaction Effect Analysis by Confidence Score

**Figure 9:**
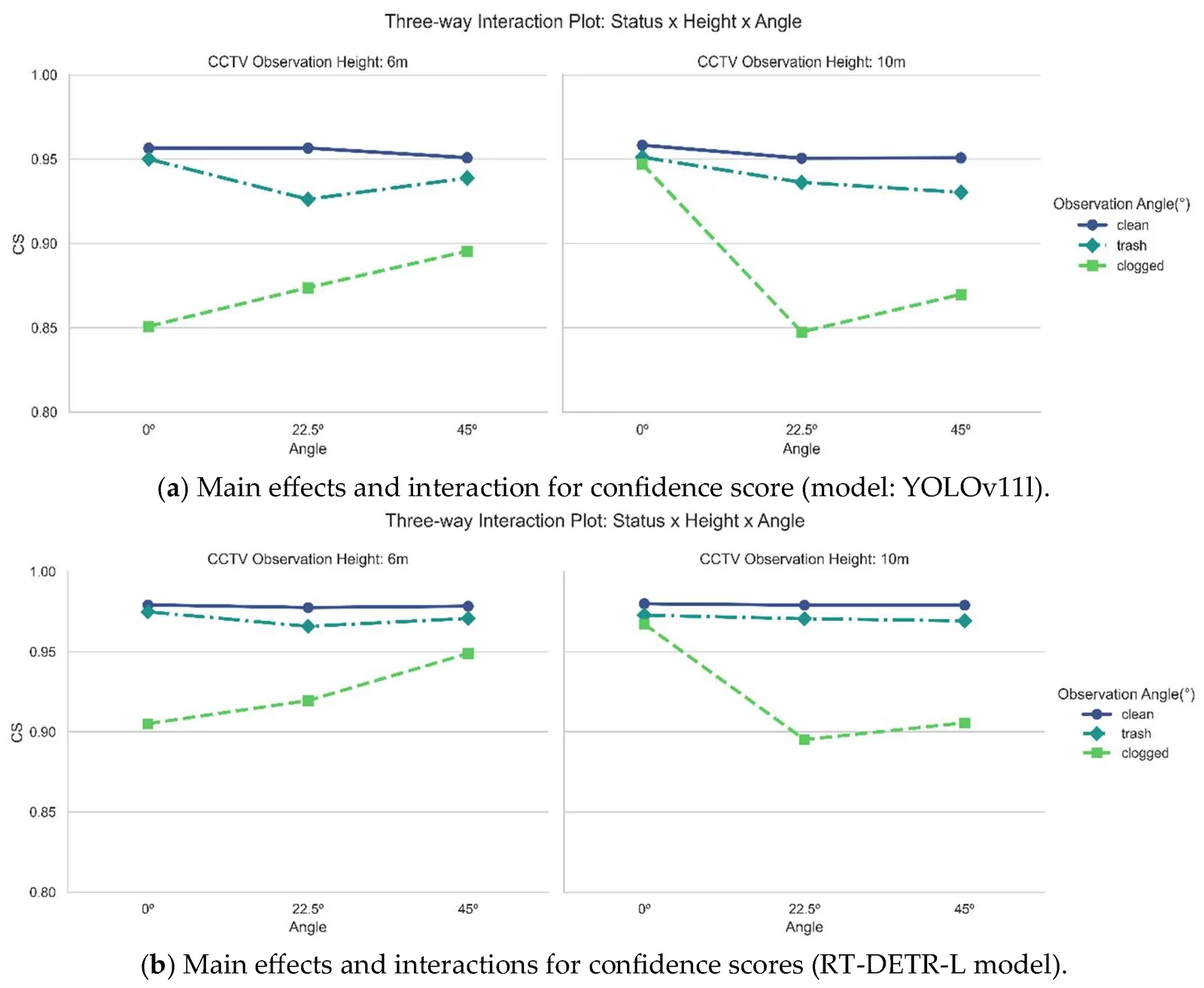
Interaction plots from the three-way ANOVA by Confidence Score.

The interaction effects of the environmental factors on the confidence scores are shown in Figure 9. Analysis of the confidence score according to the inlet condition showed that under clean and trash conditions, the confidence score remained consistently high despite changes in the experimental factors, exhibiting a stable distribution. In addition, the curves for the clean and trash conditions are nearly parallel, indicating similar response patterns. In contrast, under clogged conditions, the confidence score was relatively low and exhibited greater variability depending on the experimental conditions. At an observation height of 6 m, the confidence score under the clogged condition was the lowest at the frontal observation angle (0°) and gradually increased toward the oblique observation angles (22.5° and 45°). Conversely, at an observation height of 10 m, the highest confidence score was obtained at 0°, whereas lower values were observed at 22.5° and 45°, showing an opposite trend to that observed at 6 m.

Previous studies reported that when an object is occluded, contextual information from the surrounding environment plays an important role in object recognition [22]. Specifically, when a target object is difficult to recognize, the surrounding visual features can provide complementary information for object classification. Under the clogged condition, the higher confidence score observed at 10 m under the frontal-view condition (0°) may be associated with the wider surrounding area visible around the drainage inlet. This additional contextual information may help the model recognize the inlet when its characteristic shape is partially obscured by leaves. In contrast, under the oblique-view conditions (22.5° and 45°), the increased camera-to-inlet distance makes the drainage inlet appear smaller in the image, which may make the boundary between the leaf-covered inlet and the surrounding pavement more difficult to distinguish. These visual characteristics provide a plausible explanation for the opposite performance trends observed between the frontal and oblique viewing conditions. Further quantitative analysis of contextual regions and object scale would help verify this interpretation.

### 4.3. IoU

#### 4.3.1. Characteristics of IoU with Respect to Factor Variation

**Figure 10:**
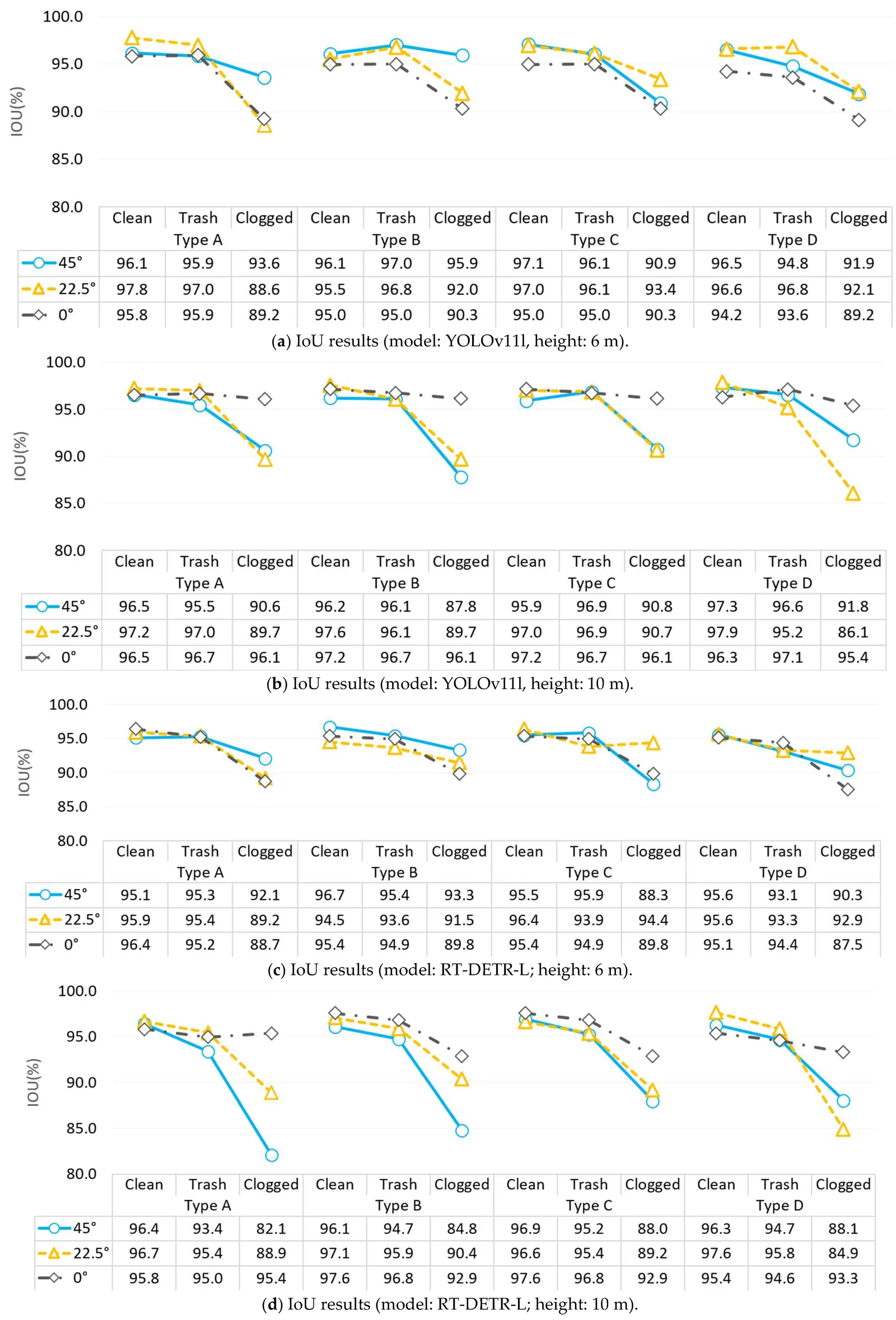
IoU results by model and observation height.

To examine how the localization accuracy of the AI models varies with external environmental factors (observation height, observation angle, and inlet condition), the IoU results are presented as percentages in Figure 10. For the clean and trash conditions, both YOLOv11l and RT-DETR-L models achieved IoU values above 90%, indicating accurate localization of the drainage inlet. However, under the clogged condition, the IoU decreased for both models, indicating that severe occlusion by leaves reduced the localization accuracy. In particular, when the inlet was observed from the front (0°), the IoU increased markedly as the observation height increased for both models. In contrast, under side-view conditions (22.5° and 45°), the IoU decreased slightly with increasing observation height. This opposite trend between the front and side observation angles is consistent with the confidence score results presented in Section 4.2.1, suggesting that the classification confidence and localization accuracy respond similarly to changes in observation conditions.

#### 4.3.2. ANOVA Results for Drainage Facility Cover Type by IoU

For the RT-DETR-L model dataset, although homoscedasticity was satisfied (Levene’s *p* = 0.503, F = 0.785), the normality assumption was violated due to severe negative skewness (Skewness = −2.227, exceeding the threshold of |Skewness| < 2.0) and a statistically significant Shapiro–Wilk test (W = 0.744, *p* = 1.649 × 10^−42^), despite the kurtosis (Kurtosis = 5.687) remaining within acceptable limits (|Kurtosis| < 7.0). Consequently, a nonparametric Kruskal–Wallis test was conducted to evaluate the effect of the Grid factor across large sample groups (N = 1440). The results revealed no statistically significant differences in IoU across cover types (F = 4.023, *p* = 0.259), with a negligible effect size (η^2^_h_ = 0.001), indicating that the Grid factor had no significant impact on the performance of the RT-DETR-L model.

For the YOLOv11l model dataset, homoscedasticity was also confirmed (Levene’s *p* = 0.613, F = 0.603). However, similar to the RT-DETR-L model, the normality assumption was violated due to substantial negative skewness (Skewness = −2.088, exceeding |Skewness| < 2.0) and a significant Shapiro–Wilk test (W = 0.754, *p* = 7.040 × 10^−42^), despite a moderate kurtosis (Kurtosis = 4.669). Therefore, a nonparametric Kruskal–Wallis test was applied to the dataset (N = 1440). The analysis revealed statistically significant differences in IoU across cover types (F = 10.862, *p* = 0.012), although the effect size (η^2^_h_ = 0.005) remained negligible. To identify which specific cover-type pairs contributed to this omnibus difference, a post hoc Dunn’s test with Bonferroni correction was conducted. None of the six pairwise comparisons among the four cover types (A, B, C, D) reached statistical significance after correction (all adjusted *p* = 1.000). This dissociation—a significant omnibus Kruskal–Wallis result without any significant pairwise contrast after correction—suggests that the omnibus significance likely reflects a diffuse combination of small, non-specific differences distributed across all four groups rather than a distinguishable difference between any particular pair, which is consistent with the negligible effect size.

Table 9 summarizes these results for both models. Whereas the Grid effect was not statistically significant for RT-DETR-L, it reached significance for YOLOv11l; however, the corresponding effect size was negligible (η^2^_h_ = 0.005), and post hoc testing identified no specific cover-type pair as the source of this difference. Taken together, cover type did not meaningfully affect IoU for either model in practical terms.

#### 4.3.3. Statistics by Factor by IoU

Aggregating the IoU values obtained across all observation conditions for the YOLOv11l model yielded an overall mean IoU value of 0.947. The mean IoUs for the inlet condition were 0.965, 0.961, and 0.916 for the clean, trash, and clogged conditions, respectively. Although the mean IoU for all conditions substantially exceeded the 0.5 threshold for successful object detection, the IoU was relatively lower for the clogged condition than for the clean and trash conditions because the leaves obscured the shape and boundary of the drainage facility. Descriptive statistics of IoU across observation heights and inlet conditions are presented in Table 10. For clean and trash conditions, both YOLOv11l and RT-DETR-L models achieved high mean IoUs (>94%) with minimal standard deviations, indicating consistent boundary estimation regardless of height. Under clogged conditions, overall IoU performance decreased and variability widened. However, in this clogged state, YOLOv11l demonstrated a higher performance floor and ceiling than RT-DETR-L. These results suggest that while both models handle unobstructed inputs well, YOLOv11l maintains superior stability in localization accuracy under adverse inlet conditions.

The descriptive statistics of the IoU by observation angle are presented in Table 11. For clean and trash conditions, both YOLOv11l and RT-DETR-L models achieved a mean IoU above 94%, with a standard deviation below 1.1%, indicating a stable IoU across the observation angles. For the clogged condition, the mean IoU was above 88%, with a standard deviation below 3.4%, indicating a lower and less stable IoU than that for the clean and trash conditions. Comparing the model performance under clogged conditions, the mean IoU of the YOLOv11l model was approximately 0.1–3.2 percentage points higher than that of the RT-DETR-L model across all observation angles, indicating a superior performance for the YOLOv11l model. However, the standard deviation varied by angle, with the RT-DETR-L model showing greater stability at 0° and the YOLOv11l model showing greater stability at 22.5° and 45°. Therefore, no consistent difference in variability between the models was observed.

#### 4.3.4. Three-Way ANOVA Results by IoU

The results of three-way ANOVA are presented in Table 12. To evaluate the IoU scores of RT-DETR-L, assumption tests for normality and homogeneity of variance were conducted across four candidate transformations. The raw IoU data violated both normality (Shapiro–Wilk W = 0.825, *p* < 0.001) and homoscedasticity (Levene’s F = 39.116, *p* < 0.001). Neither the logarithmic (W = 0.796, F = 38.207) nor the square-root transformation (W = 0.811, F = 38.724) meaningfully improved these violations. The Box–Cox transformation substantially improved normality (Shapiro–Wilk W = 0.990, *p* < 0.001), formally classified as satisfied, whereas homoscedasticity remained statistically violated (Levene’s F = 5.830, *p* < 0.001), though markedly reduced relative to the untransformed data. Accordingly, the Box–Cox-transformed IoU scores were adopted for the subsequent three-way ANOVA.

To evaluate the IoU scores of YOLOv11l, the same procedure was applied. The raw IoU data violated both normality (Shapiro–Wilk W = 0.842, *p* < 0.001) and homoscedasticity (Levene’s F = 43.162, *p* < 0.001). Logarithmic (W = 0.816, F = 42.543) and square-root transformations (W = 0.829, F = 42.915) provided negligible improvement. The Box–Cox transformation substantially improved normality (Shapiro–Wilk W = 0.991, *p* < 0.001), formally classified as satisfied, while homoscedasticity remained violated (Levene’s F = 7.034, *p* < 0.001), albeit with a considerable reduction from the raw data. The Box–Cox-transformed IoU scores were therefore adopted for the final analysis.

Three-way ANOVA revealed that YOLOv11l exhibited consistently larger main effects across all three factors (Status, Height, Angle) compared to RT-DETR-L, suggesting greater overall sensitivity to individual condition changes. However, the interaction structure differed notably between models: YOLOv11l showed a significant Status × Height interaction (*p* = 0.002) and a comparatively large Height × Angle interaction (η^2^p = 0.113), whereas RT-DETR-L’s Status × Height interaction was non-significant (*p* = 0.096), indicating that height effects in RT-DETR-L were largely independent of inlet condition. Conversely, RT-DETR-L displayed a substantially larger three-way interaction (η^2^p = 0.258 vs. 0.144), suggesting that a considerable portion of RT-DETR-L’s detection performance variability is attributable to specific combinations of status, height, and angle rather than to any single factor in isolation. While this does not establish that all three factors must be jointly considered to fully characterize the model’s behavior, it does indicate that pairwise or main-effect-based interpretations alone may be insufficient to capture RT-DETR-L’s performance patterns as completely as they do for YOLOv11l. Post hoc tests were not conducted for the three-way ANOVA because the study focused on the magnitude of main and interaction effects (η^2^p) rather than subgroup-level ranking; simple-effect analyses of the significant interactions remain a limitation.

#### 4.3.5. Interaction Effect Analysis by IoU

**Figure 11:**
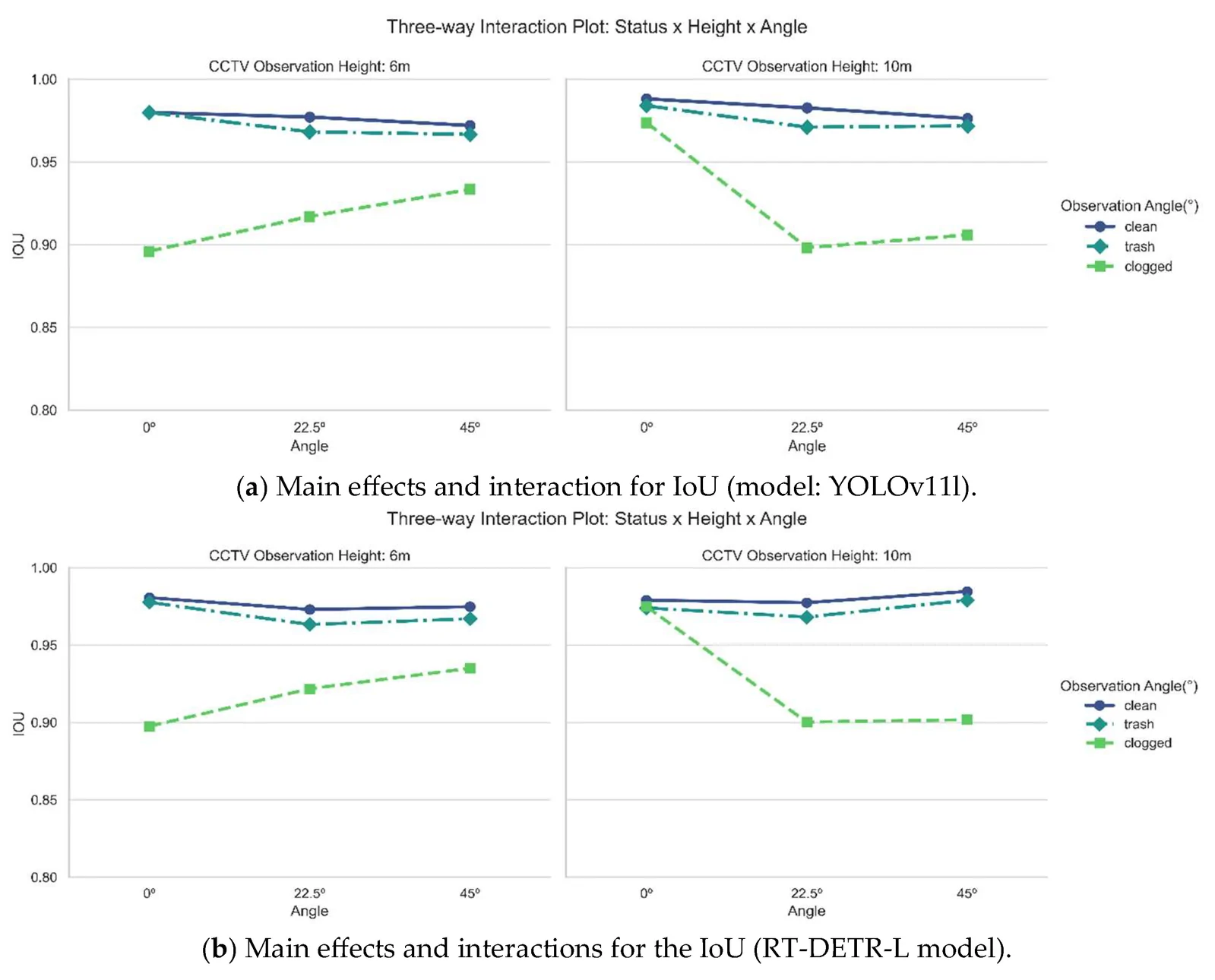
Interaction plots from the three-way ANOVA by IoU.

The variation in the IoU with respect to the inlet condition, observation height, and observation angle is shown in Figure 11. For both models, the clean and trash conditions maintained a stable, high IoU above 0.95, with little influence from the observation height or angle. In contrast, the clogged condition exhibited a relatively unstable pattern, with the IoU decreasing to approximately 0.9, which was attributed to the greater difficulty in identifying the intrinsic features of the object under the clogged condition compared to the clean and trash conditions. However, even under clogged conditions, the IoU approached the level observed for clean and trash conditions when a high observation height (10 m) was combined with a front-facing observation angle (0°). Nonetheless, under the same combination of conditions, the IoU decreased substantially when the angle changed to 22.5° or 45°, indicating that this improvement reflected a localized performance gain under a specific combination of conditions rather than genuine robustness.

## 5. Discussion

In this study, YOLOv11l and RT-DETR-L were applied to video-based automated monitoring of drainage facilities, and the quantitative and statistical effects of external environmental factors on model detection and classification performance were investigated. The academic significance and practical implications of these findings are discussed below:

### 5.1. Statistical Insignificance of Drainage Cover Type

The nonparametric tests indicated that variation in drainage cover type did not produce statistically significant differences in confidence score for either model, nor in IoU for RT-DETR-L. Although the omnibus Kruskal–Wallis test for IoU reached statistical significance for YOLOv11l, the effect size was negligible (η^2^_h_ = 0.005), and post hoc pairwise comparisons (Dunn’s test, Bonferroni-corrected) revealed no significant difference between any specific pair of cover types (all adjusted *p* = 1.000). This suggests that cover type, at least within the range examined, was not a dominant factor driving detection performance; it is possible that the models may have relied more on contextual features surrounding the drainage facility, such as the road curb and pavement, although this study did not independently quantify or verify the models’ reliance on such features, and this interpretation should be treated as a hypothesis warranting further investigation. Nonetheless, the practical implication remains that the proposed approach did not exhibit performance degradation across the drainage cover types tested, suggesting reasonable initial robustness to design variations that may exist among local governments—though broader validation across a wider range of cover designs would strengthen this claim.

### 5.2. Role of Contextual Environmental Information Under the Fully Blocked Condition

The overall model performance decreased under the fully blocked (clogged) condition because the intrinsic visual characteristics of the drainage facility, such as its rectangular outline and drainage openings, were largely obscured by the leaves. Nevertheless, the model performance improved when the observation height increased from 6 to 10 m under the front-view (0°) observation condition. This finding is consistent with that of Bell et al. [22], who reported that contextual information surrounding an occluded object plays an important role in object recognition. As the camera height increased, the image distortion near the center of the image decreased, and a broader portion of the surrounding road surface and curb became visible. Consequently, the models appeared to utilize these contextual cues to reliably infer the location of the drainage facility. Conversely, under oblique observation angles (22.5° and 45°), increasing the observation height reduced the object size within the image while simultaneously decreasing the image resolution available to distinguish the boundary between the leaf pile and the surrounding pavement. These results suggest that changes in the visible surrounding context and the apparent size of the drainage inlet may have contributed to the observed differences in confidence score and IoU. However, because these factors were not independently quantified in this study, this interpretation should be regarded as a plausible hypothesis rather than a directly verified causal mechanism.

### 5.3. Comparison of Performance Characteristics Between Detection Models

Both YOLOv11l and RT-DETR-L achieved excellent object detection performance with an identical F1 score of 0.994. However, differences emerged in their robustness under various environmental conditions. Regarding the confidence score, RT-DETR-L showed higher raw confidence scores than YOLOv11l under the tested conditions; however, because the confidence outputs of the two architectures are not necessarily calibrated on the same probabilistic scale, these values should not be interpreted as directly comparable probabilities. The difference was relatively small under the clean and trash conditions but became substantially larger under the clogged condition, reaching approximately 6 percentage points when grouped by observation height and up to approximately 7.9 percentage points when grouped by observation angle. The reanalyzed results indicate that RT-DETR-L showed greater sensitivity to observation geometry under the tested conditions, particularly for angle and interaction effects. For IoU, which reflects the localization accuracy, YOLOv11l achieved a slightly higher mean IoU than RT-DETR-L under the clogged condition across all observation angles (approximately 0.1–3.2 percentage points). However, the variability of the IoU differed depending on the observation angle, and neither model consistently outperformed the other in terms of stability. These findings suggest that YOLOv11l provides slightly more accurate localization, whereas RT-DETR-L exhibits higher raw confidence scores under severe occlusion conditions. The model inference was performed using an Intel Core i7-14700 CPU, an NVIDIA GeForce RTX 4070 Ti SUPER GPU (16 GB), and 64 GB of RAM. With an input image size of 640 × 640 pixels and a batch size of one, the end-to-end processing time per image, including preprocessing and postprocessing, ranged from approximately 16 to 28 ms for YOLOv11l and 22–38 ms for RT-DETR-L, corresponding to processing speeds of approximately 36–63 and 26–45 FPS, respectively. Accordingly, YOLOv11l’s lower per-image processing time makes it more suitable for real-time monitoring applications requiring simultaneous analysis of multiple CCTV streams, whereas the higher computational cost of RT-DETR-L may be acceptable in centralized monitoring systems where processing speed is less critical.

### 5.4. Practical Implications and Future Work

The results discussed above show that, under the clogged condition, the effect of observation height depends on observation angle. Within the conditions tested in this study, increasing the observation height from 6 to 10 m improved both confidence score and IoU at the vertical-view condition (0°), whereas the same improvement was not observed at the oblique-view conditions (22.5° and 45°). This result should not be interpreted as a general recommendation for a 10 m installation height. Rather, it indicates that observation height and angle should be considered together when evaluating CCTV configurations for drainage inlet monitoring.

The practical implications differ for new and existing CCTV systems. For new installations intended to include drainage inlet monitoring, camera location, installation height, and viewing direction can be considered together at the planning stage. For existing CCTV systems, where the camera position and viewing direction are already fixed, the suitability of the available footage should first be assessed considering the installation height, viewing angle, and resulting image scale of the drainage inlet. For oblique-view footage, perspective correction or AI-based super-resolution may be examined in future work. These preprocessing methods were not evaluated in this study, and their effects on detection performance need to be verified using actual CCTV footage.

The findings should be interpreted within the scope of the experiment. Only two observation heights (6 and 10 m) and three observation angles (0°, 22.5°, and 45°) were evaluated, and both the training and evaluation images were obtained from the same controlled testbed and video acquisition environment. Accordingly, the high detection performance reported in this study should be interpreted as same-scene, in-domain performance and should not be regarded as evidence of generalization to independent field environments. The blockage conditions were reproduced using fallen leaves only, and the partially blocked condition was not defined using a predetermined quantitative blockage ratio. Rainfall, snowfall, nighttime illumination, shadows, and occlusion by vehicles or pedestrians were also not included in the experiment.

Future work should validate the proposed approach using independent CCTV data collected at different sites and times under actual roadside conditions. This validation should cover a wider range of installation heights and observation angles, diverse environmental and traffic conditions, and debris types other than fallen leaves. The partial-blockage condition should also be examined quantitatively by estimating the blockage ratio from the proportion of the drainage inlet area covered by debris and evaluating model performance across multiple blockage levels.

## 6. Conclusions

In this study, a CCTV-based automated monitoring technology that can automatically detect and classify the blockage conditions of road drainage inlets using AI models was developed. A testbed was established by systematically varying the drainage inlet condition, observation height, and observation angle, which are considered key factors affecting object detection performance in CCTV-based monitoring environments. Application of the collected dataset to the YOLOv11l and RT-DETR-L models showed that both models achieved an F1 score of 0.994, demonstrating the feasibility of AI-based object detection and condition assessment of road drainage facilities using CCTV imagery. To quantitatively evaluate the model performance under varying environmental conditions, descriptive statistical analyses and statistical hypothesis tests were conducted using confidence scores, representing the confidence of the model prediction, and the IoU, representing the localization accuracy. Both the confidence score and IoU decreased as the inlet condition changed from clean to trash and finally to clogged, indicating that increasing the visual occlusion reduced the model performance. Under clogged conditions, both models exhibited improved confidence scores and IoU with increasing observation height when observed from the front (0°), whereas the opposite trend was observed at oblique observation angles (22.5° and 45°). Nonparametric tests further demonstrated that drainage cover type did not significantly affect confidence score for either model or IoU for RT-DETR-L; although a statistically significant, but negligible and non-specific, effect of cover type on IoU emerged for YOLOv11l. RT-DETR-L showed higher raw confidence scores than YOLOv11l, with the largest difference occurring under the clogged condition, although RT-DETR-L was more sensitive to observation angle for confidence score, whereas YOLOv11l was more sensitive to height and angle for the IoU main effects; YOLOv11l achieved a slightly higher mean IoU than RT-DETR-L under the clogged condition, although the model that showed greater variability (standard deviation) differed depending on the observation angle. Three-way ANOVA demonstrated that the inlet condition was the most influential factor affecting both the confidence score and IoU. Although the observation height and observation angle showed statistically significant main effects, their influence became particularly pronounced under clogged conditions, in which the model performance depended strongly on the interaction between the observation height and observation angle. Similarly, the interaction analysis showed that the clogged condition produced a lower confidence score and IoU, together with greater variability, than the clean and trash conditions, indicating that object recognition under severe occlusion was considerably more sensitive to environmental observation conditions.

Overall, the inlet condition was identified as the dominant factor governing both prediction confidence and localization accuracy. Under clogged conditions, where the intrinsic geometric characteristics of the drainage inlet are largely obscured, AI models appear to rely more heavily on contextual information from the surrounding environment, whose availability varies based on the combination of the observation height and observation angle. These testbed findings offer practical insights into model selection based on operational priorities, as well as considerations for evaluating CCTV configurations in terms of installation height and observation angle. However, these findings are based on evaluation under controlled environmental conditions with fixed facility locations and clear weather. To bridge the gap between testbed validation and practical implementation, independent field validation is required. Future studies must evaluate and refine the proposed framework using diverse real-world datasets captured under unconstrained conditions, including rain, snow, nighttime illumination, and dynamic occlusions caused by vehicles or pedestrians. Upon successful field validation, the proposed technology holds strong potential for integration with existing municipal security or multipurpose CCTV infrastructures. Ultimately, the future deployment of edge devices transmitting real-time detection results could assist municipal monitoring centers in identifying blocked drainage facilities more rapidly, thereby supporting urban flood prevention and mitigation efforts. This study had several limitations. The evaluation was conducted under controlled environmental conditions with fixed drainage facility locations and clear weather.

The proposed CCTV-based drainage facility monitoring approach has potential for future integration with existing municipal security or multipurpose CCTV infrastructures, subject to further validation using independent field data. The combined effect of observation height and angle identified in this study also suggests that these two factors should be considered jointly when evaluating CCTV configurations for drainage inlet monitoring. Further validation using independent CCTV data collected from diverse real-world roadside environments is needed to extend the practical applicability of the present findings. Future studies should validate the proposed framework using independent datasets collected from diverse installation sites under varying environmental conditions, including rainfall, snowfall, nighttime illumination, and occlusion by vehicles or pedestrians. The initial applicability of the proposed models can be further enhanced by incorporating footage from existing security and traffic-monitoring CCTV systems operating under diverse conditions into the training dataset.

## Figures and Tables

**Table 1:** Comparison with Previous Studies.

Study	Research Content	Data Scale	Environmental Conditions	Differentiation from This Study
Pichaikutty [5]	Faster R-CNN-based automatic detection of curbside storm drains using Google Street View imagery	900 images selected for the dataset 1000 test images for model validation (600 storm drains)	Google Street View (GSV)-based ground-level imagery of roads and highways (640 × 640 px)	Limited to storm drain localization; unable to determine inlet blockage status
Commandre et al. [6]	CNN-based (custom AlexNet) automatic manhole cover detection and localization using aerial imagery	18,405 manhole images and 458,915 other-object images after data augmentation	Very high-resolution (VHR; 5 cm resolution) natural-color (RGB) aerial remote sensing imagery	Limited applicability to ground-level CCTV environments
Pang et al. [7]	Development of a fast, lightweight real-time detection algorithm (MGB-YOLO) for road manhole covers in a vehicle-embedded environment	1000 original road-surface images 6000 images after data augmentation	Manhole cover detection using a vehicle-mounted device	Based on a vehicle-driving viewpoint, differing in field of view (FOV) from a fixed roadside CCTV environment
Yin et al. [8]	Development of an algorithm based on a deformable attention multimodal network (DAMNet) fusing RGB imagery and LiDAR point-cloud data for road and water-surface object detection	KITTI Dataset: 14,996 frames (7481 training/7515 testing)	Challenging conditions including reflective surfaces, low-texture environments, and varying lighting conditions	This study targets small-object detection in marine/water-surface environments using sensor fusion (RGB + LiDAR)
Ge et al. [9]	Development of a framework using digital surface models (DSM) and grid-based line-of-sight simulation to evaluate CCTV camera detection and positioning performance for C-V2X applications	3D terrain and LiDAR data collected from one freeway site at DataCity Smart Mobility Testing Ground (New Brunswick, NJ, USA)	Varying environmental and camera configuration scenarios Challenges with object scales, aspect ratios, and viewing perspectives in existing surveillance environments	Focuses on evaluating camera placement and positioning performance for vehicle/pedestrian tracking in C-V2X
This Study	Development of an AI model (YOLOv11l, RT-DETR-L)-based algorithm for classifying drainage inlet blockage status (3 categories) using CCTV imagery	38,239 frame-level images extracted from video footage 1440 representative images selected while minimizing visual redundancy (20 images per scenario) Random 8:2 split into training and evaluation datasets	Fixed CCTV footage from a demonstration center testbed Camera height (6 m, 10 m), observation angle (0°, 22.5°, 45°) 4 drainage cover types and 3 inlet blockage condition scenarios	–

**Table 2:** Example images by CCTV observation height and angle.

Angle	Height	
	6 m	10 m
0°	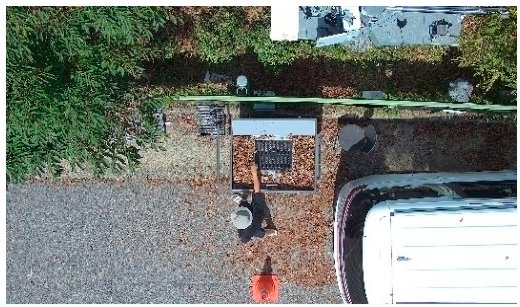	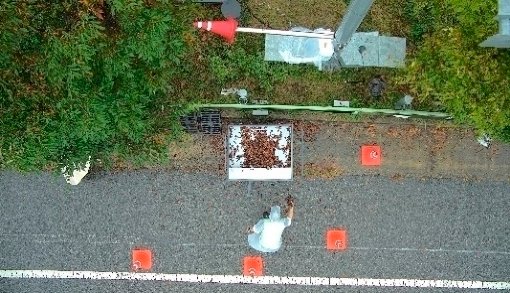
22.5°	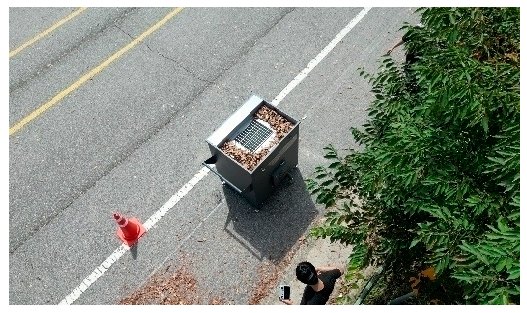	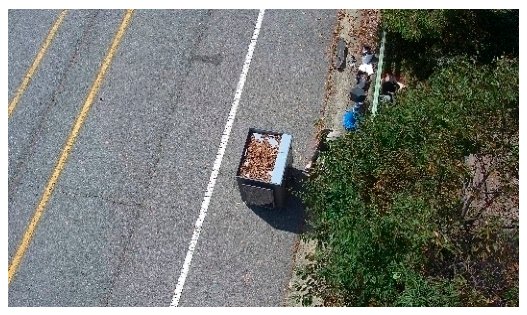
45°	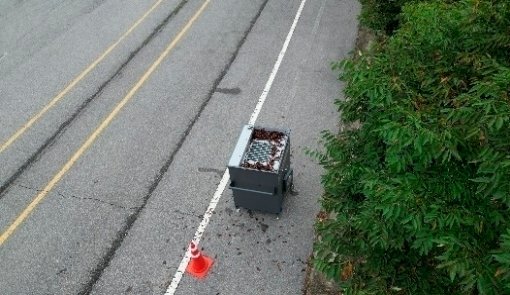	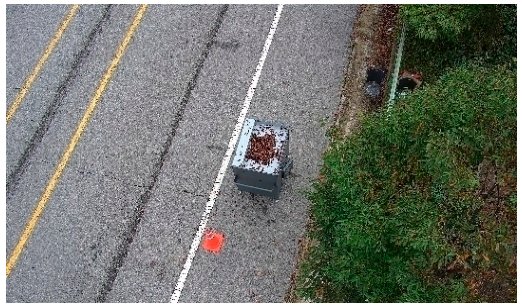

**Table 3:** Results of object detection model analysis (example).

**Cover Type**	**Drainage Inlet Condition (Observation Angle)**		
	**Clean (45°)**	**Trash (22.5°)**	**Clogged (0°)**
Grid type	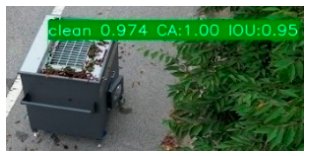	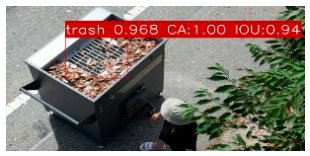	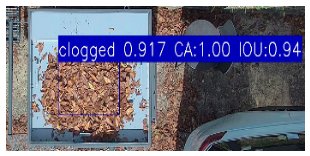
Transverse type A	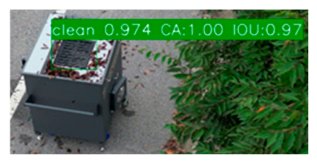	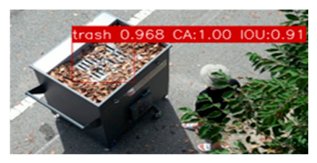	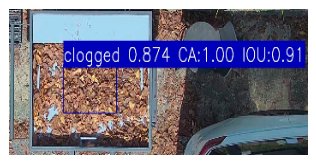
Transverse type B	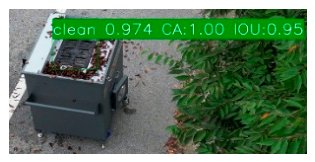	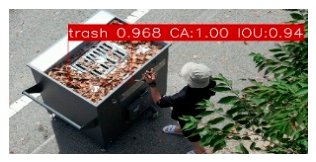	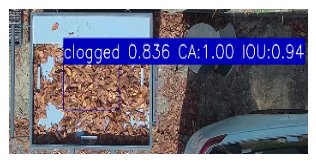
Transverse type C	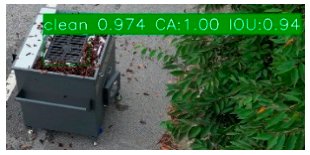	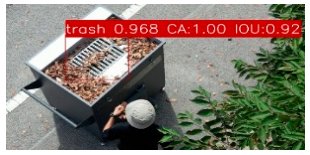	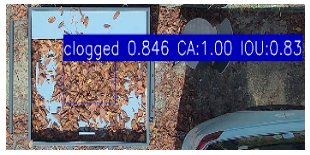

**Table 4:** Object detection performance of YOLOv11l and RT-DETR-L.

Model	Class	Precision	Recall	F1 Score
YOLOv11l	Clean	0.994	0.998	0.996
	Trash	0.992	0.997	0.994
	Clogged	0.990	0.996	0.993
	Mean	0.992	0.997	0.994
RT-DETR-L	Clean	0.995	0.997	0.996
	Trash	0.993	0.996	0.994
	Clogged	0.991	0.995	0.993
	Mean	0.993	0.996	0.994

**Table 5:** Statistical comparisons for confidence score by drainage facility cover type.

Model	Source	df	F(H)	*p*-Value	Effect Size
YOLOv11l	Grid	3	2.378	0.498	0.000
	Residual	1436	-	-	
RT-DETR-L	Grid	3	0.663	0.575	0.001
	Residual	1436	-	-	

**Table 6:** Descriptive statistics of confidence score by model and observation height.

Height	Condition	YOLOv11l				RT-DETR-L			
		Average	Std.	Max.	Min.	Average	Std.	Max.	Min.
6 m	Clean	94.2	0.4	94.8	93.5	97.5	0.1	97.7	97.3
	Trash	94.3	0.5	95.5	93.7	97.0	0.3	97.4	96.7
	Clogged	87.6	2.2	91.3	83.9	93.6	1.6	95.9	91.3
10 m	Clean	94.5	0.4	95.1	94	97.6	0.1	97.7	97.4
	Trash	94.2	0.4	94.8	93.6	96.9	0.4	97.5	96.3
	Clogged	87.2	4.3	93.7	82.9	93.7	2.5	97.4	90.6

**Table 7:** Descriptive statistics of confidence score by model and observation angle.

Angle	Condition	YOLOv11l				RT-DETR-L			
		Average	Std.	Max.	Min.	Average	Std.	Max.	Min.
0°	Clean	94.9	0.2	95.1	94.4	97.7	0.1	97.7	97.6
	Trash	94.7	0.4	95.5	94.3	97.3	0.1	97.5	97.1
	Clogged	89.6	3.9	93.7	83.9	94.4	2.8	97.4	91.3
22.5°	Clean	94.1	0.3	94.5	93.5	97.5	0.1	97.6	97.3
	Trash	94.1	0.4	94.8	93.6	96.7	0.1	96.9	96.5
	Clogged	85.5	1.9	89.6	83.5	93.4	1.3	95.9	91.6
45°	Clean	94.1	0.2	94.5	93.8	97.4	0.1	97.6	97.3
	Trash	93.9	0.2	94.1	93.7	96.8	0.2	97.1	96.3
	Clogged	87.2	2.8	91.3	82.9	93.2	1.8	95.8	90.6

**Table 8:** Three-way ANOVA results for confidence score.

Source	YOLOv11l				RT-DETR-L			
	df	F	*p*-Value	Effect Size	df	F	*p*-Value	Effect Size
Status (Inlet Condition)	2	887.346	<0.001	0.555	2	23,123.283	<0.001	0.970
Height	1	12.538	<0.001	0.009	1	207.539	<0.001	0.127
Angle	2	67.872	<0.001	0.087	2	932.859	<0.001	0.567
Status:Height	2	10.051	<0.001	0.014	2	64.237	<0.001	0.083
Status:Angle	4	16.524	<0.001	0.044	4	167.408	<0.001	0.320
Height:Angle	2	89.504	<0.001	0.112	2	718.089	<0.001	0.502
Status:Height:Angle	4	87.705	<0.001	0.198	4	927.171	<0.001	0.723

**Table 9:** Statistical comparisons for IoU by drainage facility cover type.

Model	Source	df	F	*p*-Value	Effect Size
YOLOv11l	Grid	3	10.862	0.012	0.005
	Residual	1436	-	-	
RT-DETR-L	Grid	3	4.023	0.259	0.001
	Residual	1436	-	-	

**Table 10:** Descriptive statistics of IoU by model and observation height.

Height	Condition	YOLOv11l				RT-DETR-L			
		Average	Std.	Max.	Min.	Average	Std.	Max.	Min.
6 m	Clean	96.0	1.0	97.8	94.2	95.6	0.6	96.7	94.5
	Trash	95.8	1.0	97.0	93.6	94.6	0.9	95.9	93.1
	Clogged	91.4	2.1	95.9	88.6	90.7	2.1	94.4	87.5
10 m	Clean	96.9	0.6	97.9	95.9	96.7	0.7	97.6	95.4
	Trash	96.4	0.6	97.1	95.2	95.3	0.9	96.8	93.4
	Clogged	91.7	3.3	96.1	86.1	89.2	3.8	95.4	82.1

**Table 11:** Descriptive statistics of IoU by model and observation angle.

Angle	Condition	YOLOv11l				RT-DETR-L			
		Average	Std.	Max.	Min.	Average	Std.	Max.	Min.
0°	Clean	95.9	1.0	97.2	94.2	96.1	0.9	97.6	95.1
	Trash	95.9	1.1	97.1	93.6	95.3	0.9	96.8	94.4
	Clogged	92.8	3.1	96.1	89.2	91.3	2.5	95.4	87.5
22.5°	Clean	97.1	0.7	97.9	95.5	96.3	0.9	97.6	94.5
	Trash	96.5	0.6	97.0	95.2	94.8	1.0	95.9	93.3
	Clogged	90.3	2.2	93.4	86.1	90.2	2.7	94.4	84.9
45°	Clean	96.5	0.5	97.3	95.9	96.1	0.6	96.9	95.1
	Trash	96.1	0.7	97.0	94.8	94.7	0.9	95.9	93.1
	Clogged	91.6	2.2	95.9	87.8	88.4	3.4	93.3	82.1

**Table 12:** Three-way ANOVA results by IoU.

Source	YOLOv11l				RT-DETR-L			
	df	F	*p*-Value	Effect Size	df	F	*p*-Value	Effect Size
Status (Inlet Condition)	2	1171.820	<0.001	0.622	2	931.937	<0.001	0.567
Height	1	130.822	<0.001	0.084	1	72.736	<0.001	0.049
Angle	2	170.809	<0.001	0.194	2	90.389	<0.001	0.113
Status:Height	2	6.264	0.002	0.009	2	2.346	0.096	0.003
Status:Angle	4	14.104	<0.001	0.038	4	12.667	<0.001	0.034
Height:Angle	2	90.456	<0.001	0.113	2	26.591	<0.001	0.036
Status:Height:Angle	4	59.808	<0.001	0.144	4	123.386	<0.001	0.258

## Data Availability

The data presented in this study are available on request from the corresponding author.
